# The clinical–epidemiological profile of malaria patients from Southern Venezuela, a critical hotspot in Latin America

**DOI:** 10.1186/s12936-021-03913-w

**Published:** 2021-09-20

**Authors:** David A. Forero-Peña, Fhabián S. Carrión-Nessi, Melynar Chavero, Ángel Gamardo, Luisamy Figuera, Natasha A. Camejo-Ávila, María V. Marcano, Mariana Hidalgo, Cariagne J. Arenas-Leal, Leopoldo Villegas, María E. Grillet, M. Andreína Pacheco, Marisol Sandoval-de Mora, Ananías A. Escalante

**Affiliations:** 1 Biomedical Research and Therapeutic Vaccines Institute, Ciudad Bolivar, Venezuela; 2 Department of Internal Medicine, “Ruiz Y Páez” University Hospital Complex, Ciudad Bolivar, Venezuela; 3 “Dr. Francisco Battistini Casalta” Health Sciences School, University of Oriente – Bolivar Nucleus, Ciudad Bolivar, Venezuela; 4 Venezuelan Institute of Scientific Research (IVIC), Miranda, Venezuela; 5 Civil Association of Social Impact (ASOCIS), Tumeremo, Venezuela; 6 Global Development One (GDO), Maryland, USA; 7 Vector and Parasite Biology Laboratory, Tropical Ecology and Zoology Institute, Faculty of Sciences, Central University of Venezuela, Caracas, Venezuela; 8 Biology Department/Institute of Genomics and Evolutionary Medicine (iGEM), Temple University, Philadelphia, PA 19122-1801 USA

**Keywords:** Severe malaria, Plasmodium vivax, Plasmodium falciparum, Cerebral malaria

## Abstract

**Background:**

Venezuela accounted for 55% of the cases and 73% of the malaria deaths in the Americas in 2019. Bolivar state, in the southeast, contributes > 60% of the country's *Plasmodium vivax* and *Plasmodium falciparum* cases every year. This study describes the clinical–epidemiological characteristics of clinical malaria patients in this high-transmission area.

**Methods:**

A prospective study was conducted on patients seeking medical attention in three medical centres in the state capital, Ciudad Bolivar, between June and October 2018. Malaria diagnosis was carried out using microscopy following national standards. Malaria-positive patients were examined for clinical symptoms, and haematological tests were performed at the time of diagnosis. Patients were followed up by telephone to evaluate malaria recurrences.

**Results:**

Out of 287 patients, 200 (69.7%) were positive for *P. vivax*, 69 (24%) for *P. falciparum*, and 18 (6.3%) had mixed (*P. vivax*/*P. falciparum*) infections. Patients' median age was 33 years (IQR 20), 168 (69%) were men, and 40% practiced gold mining as the main occupation. Fever (96.5%), chills (91.3%), and headaches (90.6%) were the most frequent symptoms. At least one symptom associated with severe malaria was observed in 69 out of 161 patients with complete clinical evaluation (42.9%). *Plasmodium vivax* infections were found in 42 out of 69 (60.9%) severe cases; by contrast, *P. falciparum* and mixed malaria caused 34.8% (24/69) and 4.4% (3/69) of infections, respectively. Two patients died of cerebral malaria. Mean hemoglobin was lower in the patients infected with *P. falciparum* than those infected with * P. vivax*. Regardless of the parasite causing the infection, patients presented high levels of total bilirubin, aminotransferases (AST, ALT), and lactate dehydrogenase (LDH). Out of the 142 patients followed up by phone for three months (49.5% of the 287 patients), 35 (24.7%) reported recurrences.

**Conclusions:**

The high malaria prevalence among young male adults practicing gold mining suggests that this occupation is a significant risk factor. The unexpected high prevalence of *P. vivax* patients with at least one criteria of severe clinical disease is a matter of concern. Whether it is the result of a lack of timely diagnosis and effective treatment should be explored.

## Background

Malaria remains a significant public health problem worldwide [1]. There were ~ 899,000 (95% confidence interval: 822,000–970,000) estimated cases in Latin America in 2019, equivalent to ~ 0.4% of the global burden [1]. Still, the region has ~ 139 million inhabitants at risk of infection. Although several Latin American countries are on track toward malaria elimination, there has been an increase in regional cases [2–6]. Three countries accounted for almost 90% of all reported cases in 2019: Venezuela (55%), Brazil (22%), and Colombia (11%) [1]. Venezuela had an estimated 467,421 cases, with 403 fatal cases representing 73% of the Americas' malaria-related deaths [1].

Historically, Venezuela had the highest malaria incidence rate in Latin America by 1931. The situation changed dramatically in 1961. After intensive control efforts, this nation became the first World Health Organization-certified (WHO) country that eliminated malaria in almost 70% of its territory [7]. However, in Bolivar state in the southeastern region bordering Brazil and Guyana, there has been a high risk of malaria infection for decades [4]. Due to the ongoing Venezuelan health crisis, the Bolivar state has also become the hottest malaria hotspot for the Americas region [5].

The reported data from Venezuela in 2019 indicate that, like in other countries from the Americas [8–11], *Plasmodium vivax* accounted for 77.3% of reported cases, followed by *Plasmodium falciparum* (16.2%), and mixed (*P. vivax*/*P. falciparum*) malaria (6.5%) [1]. Fortunately, the frequency of mutations related to anti-malarial drug resistance in *P. falciparum* seems to remain the same regardless of the dramatic increase in transmission during the last decade [6]. However, there is limited information on patients' clinical–epidemiological characteristics during the ongoing Venezuelan malaria crisis. This lack of recent clinical data is due in part to the difficult conditions in the area. Thus, this study describes the clinical–epidemiological profile of patients seeking medical attention and diagnosed with malaria in the diagnostic centres of Ciudad Bolivar, the capital of Bolivar state, Venezuela.

## Methods

### Study site

**Fig. 1:**
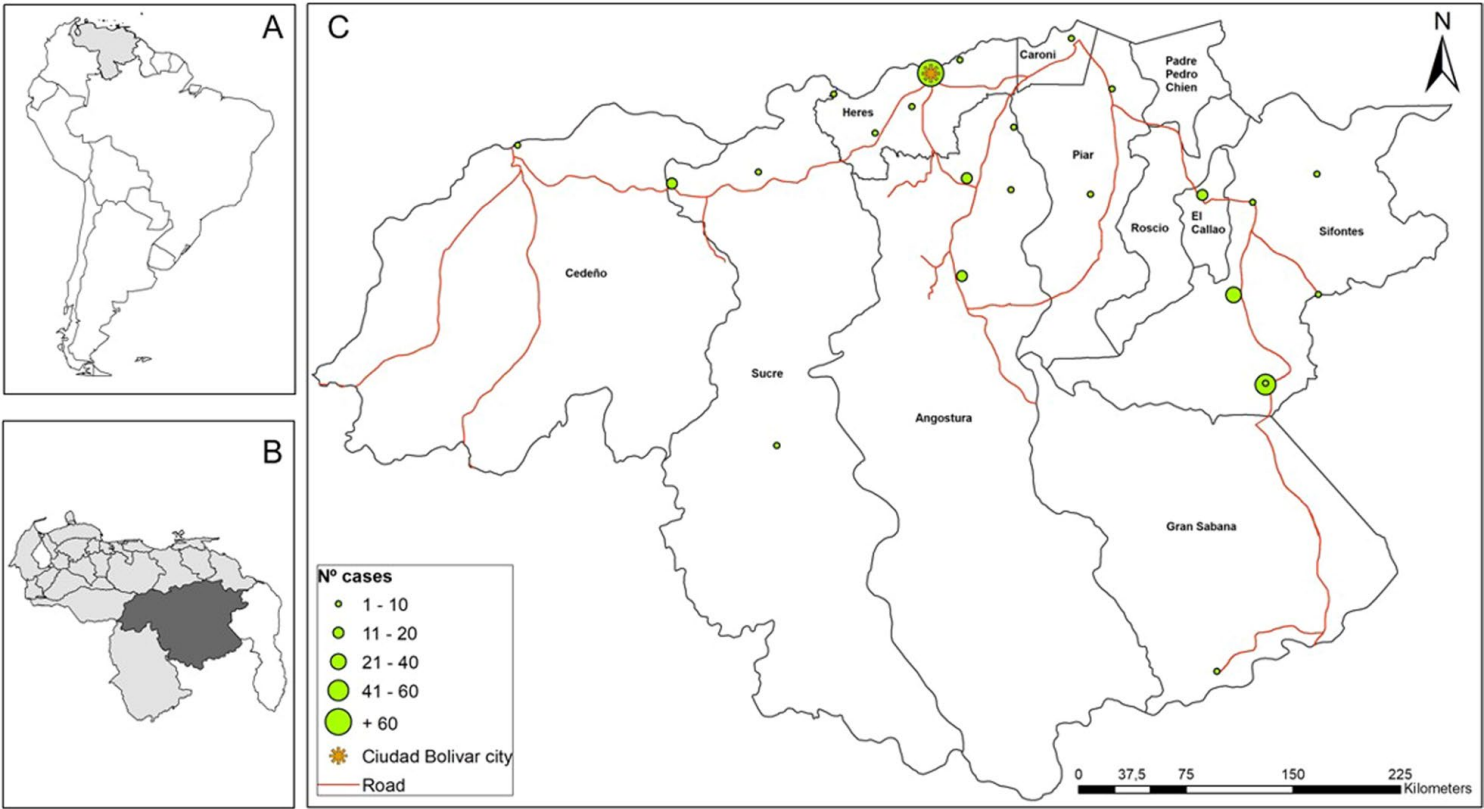
Malaria cases by locality of origin of the infection. **A** Map of Venezuela in South America (light grey); **B** the study area (Bolivar state in the south of Venezuela, dark gray); and **C** map of Bolivar state showing the malaria cases quantified according to the patients diagnosed in Ciudad Bolivar by locality of origin of the infection. The localization of the state capital, Ciudad Bolivar, is shown

Bolivar state is in the country's south, bordering Brazil and Guyana; it has 240,528 km^2^, corresponding to 26.2% of the national territory, with an approximate population of 1,837,485 inhabitants projected by 2018 [12]. *Plasmodium vivax* is the most prevalent parasite, with 70–80% of malaria cases, whereas *P. falciparum* causes the remaining 20–30% of cases annually [5]. This study was carried out in Ciudad Bolívar, the state capital (Fig. 1), with approximately 427,399 inhabitants projected by 2018 [12]. Three medical centres were selected. Those centres provide care, diagnosis, and treatment to malaria patients covering almost all the cases in the city. Specifically, samples were collected in two type-II urban outpatient health centres ("*El Perú*" and "*La Sabanita*"); these are centres without hospitalization areas attended by a general or family doctor with experience in public health administration. The third was a type IV Hospital ("*Ruíz y Páez*" University Hospital Complex), the main hospitalization centre for malaria patients in Ciudad Bolivar.

### Ethical aspects

The study protocol was reviewed and approved by the Bioethics and Health Biosecurity Committee of "Ruíz y Páez" University Hospital Complex (CHRRP-CBBS-001-2018). The study was carried out according to the ethical principles for medical research in humans of the Declaration of Helsinki and the Venezuelan regulations for this type of research, with all patients' corresponding signed informed consent.

### Study design and participants

A prospective study was conducted during the period June–October 2018. Patients positive for a malaria infection confirmed by microscopy were invited to participate. These patients sought malaria diagnosis and treatment at any of the three study sites. Trained physicians at each site performed a standard clinical evaluation and detailed physical examination of all subjects in this study. Patients were classified as non-severe or severe malaria cases according to the WHO [13] and *"Ministerio del Poder Popular para la Salud"* of Venezuela criteria [14], regardless of the malaria parasite species. All patients were treated by the local health provider, using the last national anti-malarial protocol approved (2017) by the Bolivarian Republic of Venezuela health authorities [14]. Patients with non-severe *P. vivax* malaria received chloroquine and primaquine (daily over 14 days) (*n* = 198). Given the changing conditions during the malaria epidemic, patients infected with *P. falciparum* received one of the following treatments: (a) artemether plus lumefantrine and primaquine (single dose) (*n* = 58) or (b) artesunate plus mefloquine and primaquine (single dose) (*n* = 5). Patients with mixed infections were treated with one of the following options: (a) artemether plus lumefantrine and primaquine (daily over 14 days) (*n* = 16) or (b) artesunate plus mefloquine and primaquine (daily over 14 days) (*n* = 2). Patients with severe malaria cases received (a) injectable artesunate (*n* = 6) or (b) injectable artemether (*n* = 2).

Although a total of 287 patients diagnosed with malaria accepted to participate in the study, blood samples (8 ml) by venipuncture were collected only from 161 patients due to limitations in resources. The blood samples were aliquoted into three fractions: 3 ml were preserved with sodium citrate to study coagulation (prothrombin and partial thromboplastin times), 3 ml were collected with ethylenediaminetetraacetic acid to study haematology, and 2 ml without anticoagulant were used to study blood chemistry (urea, creatinine, glycaemia, electrolytes, transaminases, lactate dehydrogenase). In this study, malaria recurrences were defined following WHO standards in terms of reappearance of parasitaemia after treatment, due to recrudescence, relapse or a new infection [15]. To estimate the frequency of recurrences, patients were followed by telephone at 15 days, one month, and three months after diagnosis. They were asked about the reappearance of parasitaemia (confirmed by microscopy) after taking treatment.

### Data analysis

Parametric tests were used, and when the assumption of normality was rejected, the data were analysed using non-parametric tests. The distribution of the parameters was statistically evaluated using Kolmogorov–Smirnov. Fisher's exact was used when necessary for variables with low frequencies. The analyses were carried out in the statistical software Statistical Package for the Social Sciences (version 25, International Business Machines Corporation, Armonk, New York, United States). Maps were performed using ArcGIS software (version 10.5.1, Redlands, California, United States: Environmental Systems Research Institute).

## Results

### Characteristics of the studied patients

**Fig. 2:**
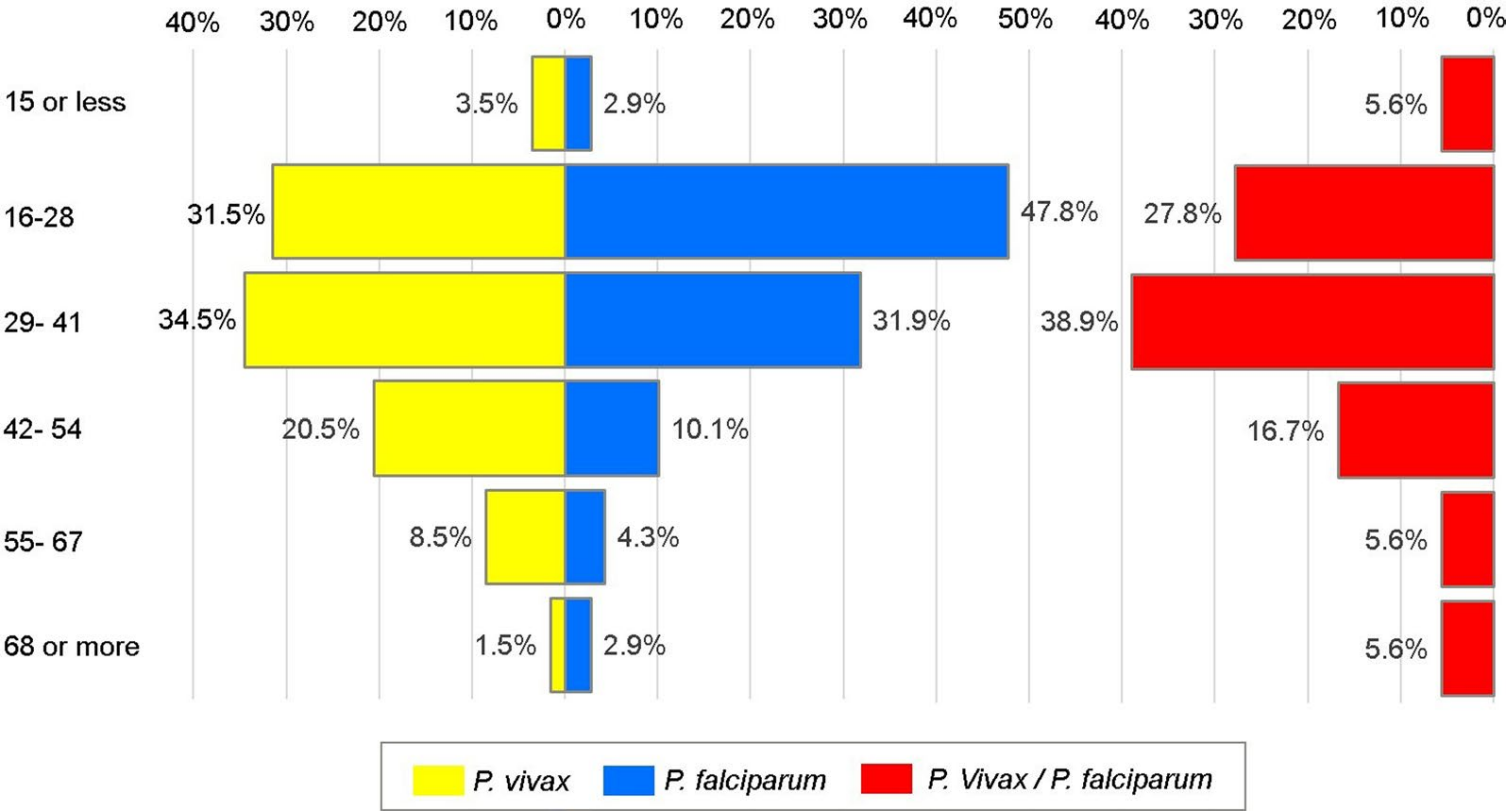
Distribution of malaria patients according to age group in Bolivar state, south of Venezuela. Bars indicate the proportion of patients according to age group and *Plasmodium* species. There were no differences between age groups and *Plasmodium* species (*p* = 0.328; Fisher's exact test)

Out of the 287 patients diagnosed with malaria who accepted participating in the study, 200 (69.7%) had *P. vivax,* 69 (24%) *P. falciparum,* and 18 (6.3%) had mixed (*P. vivax/P. falciparum*) malaria infections. The total numbers of cases by locality of origin are shown in Fig. 1. The proportion of cases by age group and parasite species is shown in Fig. 2. The patients' median age was 33 years (IQR 20). No statistical differences were observed in the age distribution between patients with *P. vivax, P. falciparum,* and mixed malaria infections (*p* = 0.12). Table 1 shows socio-demographic characteristics. Most of the patients were men (69%) working on gold mining (40.1%); 83.2% (239/287) self-reported previous malaria episodes with a median of three episodes (IQR 7). Only 22 (9.2%) of those 239 patients indicated not receiving complete anti-malarial chemotherapy when treating the last infection. The average number of days between the onset of symptoms and consultation at health facilities was 5 ± 4 days, with no significant differences between species. Only 4.5% self-reported comorbidities, such as chronic cardiovascular diseases (*n* = 8), diabetes (*n* = 3) and others.

**Table 1:** Socio-demographic characteristics of patients with malaria by *Plasmodium* species

Characteristics	Total (N = 287; 100%)	*P. vivax* (N = 200; 69.7%)	*P. falciparum* (N = 69; 24%)	Mixed malaria (N = 18; 6.3%)	*p*-value
Age, years	33 (20)	33 (19)	27 (16)	38 (25)	0.123^||^
Median (IQR)					
Min–Max	13–73	13–73	14–68	14–69	
Gender	33 (20)				0.59^†^
Male	198 (69)	135 (67.5)	51 (73.9)	12 (66.7)	
Female	89 (31)	65 (32.5)	18 (26.1)	6 (33.3)	
Race					0.14^‡^
Mestizo	269 (93.7)	191 (95.5)	61 (88.4)	17 (94.4)	
Black	11 (3.8)	6 (3)	5 (7.2)	–	
Native	7 (2.4)	3 (1.5)	3 (4.3)	1 (5.6)	
Origin by municipality					
Heres	94 (32.8)	77 (38.5)	13 (18.8)	4 (22.2)	0.01^†^
Sifontes	103 (35.9)	60 (30)	35 (50.7)	8 (44.4)	0.01^†^
Angostura	31 (10.8)	24 (12)	6 (8.7)	1 (5.6)	0.57^‡^
El Callao	17 (5.9)	11 (5.5)	3 (4.3)	3 (16.7)	0.14^‡^
Sucre	17 (5.9)	11 (5.5)	6 (8.7)	–	0.46^‡^
Piar	7 (2.3)	5 (2.5)	2 (2.9)	–	1^‡^
Cedeño	5 (1.7)	3 (1.5)	2 (2.9)	–	0.71^‡^
Caroni	3 (1)	2 (1)	1 (1.4)	–	1^‡^
Gran Sabana	2 (0.7)	1 (0.5)	–	1 (5.6)	0.17^‡^
Others	8 (2.8)	6 (3)	1 (1.4)	1 (5.6)	0.43^‡^
Scholarship					0.96^†^
None	21 (7.3)	16 (8)	4 (5.8)	1 (5.6)	
Middle school	119 (41.5)	83 (41.5)	28 (40.6)	8 (44.4)	
High school	115 (40.1)	81 (40.5)	27 (39.1)	7 (38.9)	
Technical/Professional	32 (11.1)	20 (10)	10 (14.5)	2 (11.1)	
Occupation					0.49^§^
Illegal mining	115 (40.1)	73 (36.5)	35 (50.7)	7 (38.9)	
Housewife	49 (17.1)	39 (19.5)	8 (11.6)	2 (11.1)	
Merchant	29 (10.1)	21 (10.5)	7 (10.1)	1 (5.6)	
Student	15 (5.2)	10 (5)	4 (5.8)	1 (5.6)	
Farmer	14 (4.9)	10 (5)	2 (2.9)	2 (11.1)	
Mine cook	6 (2.1)	3 (1.5)	3 (4.3)	–	
None	3 (1)	3 (1.5)	–	–	
Others	56 (19.5)	41 (20.5)	10 (14.5)	5 (27.8)	
Income/week [US dollar]	10 [65]	7.75 [65]	20 [96]	5 [73]	0.05^||^
Previous episodes of malaria [yes]	239 [83.3]	170 [85]	52 [75.4]	17 [94.4]	0.09^||^
Median of previous episodes	3 [7]	3 [5]	3 [10]	4 [8]	0.21^||^
Number of previous episodes					0.16^†^
5 or less	139 (58.2)	106 (62.4)	23 (44.2)	10 (58.8)	
Between 6 and 10	53 (22.2)	34 (20)	14 (26.9)	5 (29.4)	
11 or more	47 (19.7)	30 (17.6)	15 (28.8)	2 (11.8)	
Days to consultation	5 (4)	5 (4)	6 (5)	4 (2)	0.11^*^

The median and interquartile range (IQR) were estimated for income and a total number of previous malaria episodes, and percentage were estimated for categorical variables. *One-way ANOVA; ^†^Chi-square; ^‡^Fisher’s exact test; ^§^Yates-corrected Chi-square; ^||^Median’s test

**Fig. 3:**
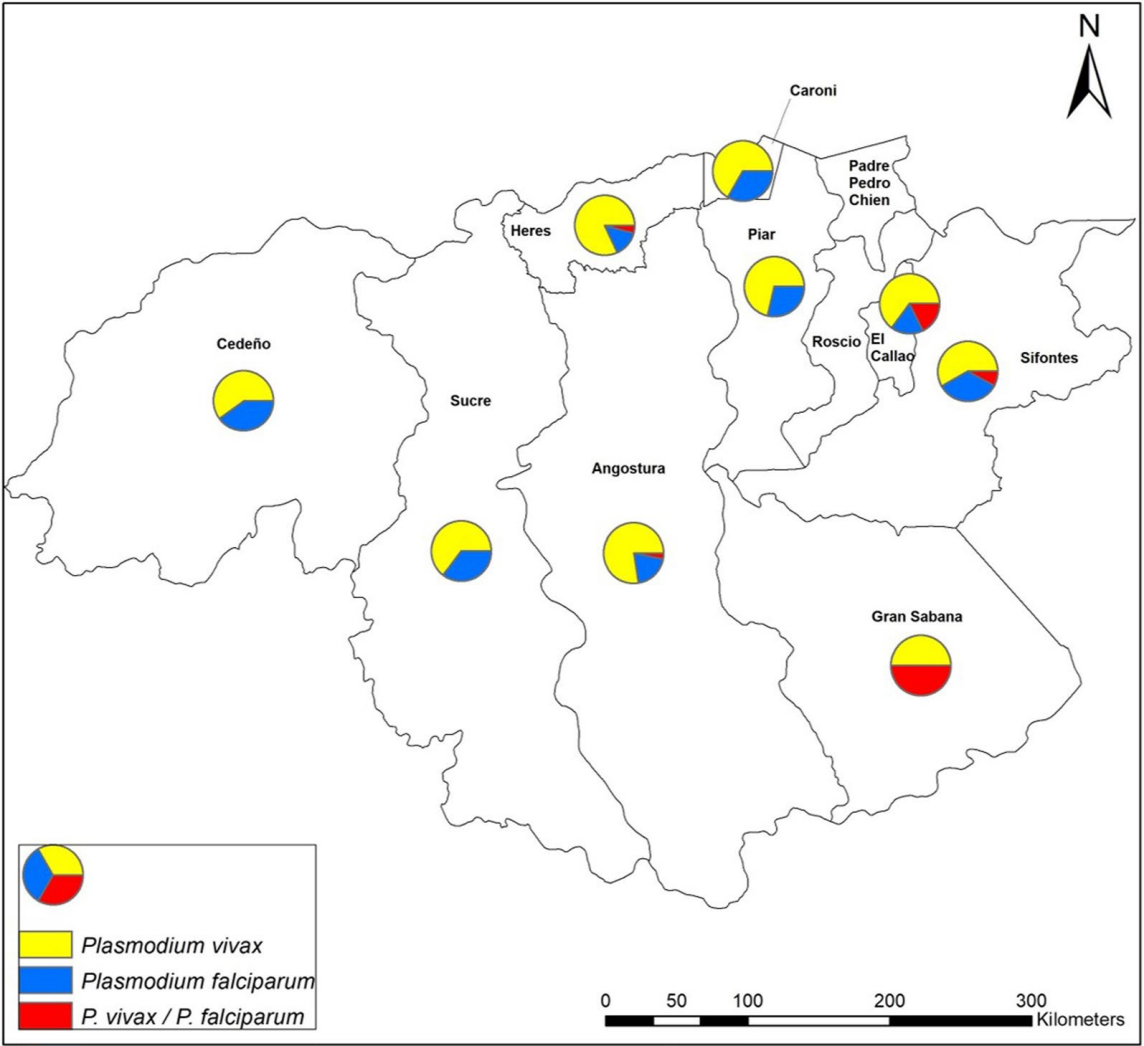
Distribution of *Plasmodium* species in Bolivar state according to the origin of patients. Pie charts indicate the proportion of *Plasmodium* species and its geographic origin (municipality) in Bolivar state from the total of patients diagnosed with malaria during 2018

Most patients (97.2%; 279/287) were from the Bolivar state. Of these, 103 (35.9%) and 94 (32.8%) came from the Sifontes and Heres municipalities, respectively. The rest of the patients were from other municipalities, such as Angostura, El Callao, Sucre, Piar, Cedeño, Caroni, and Gran Sabana (Fig. 3). A significant association was found between *P. falciparum* malaria and Sifontes' municipality's provenance (*p* = 0.01) (Fig. 3).

### Clinical findings

Table 2 summarizes the malaria symptoms and signs observed in the patients who participated in the study. No significant differences in symptoms between *P. falciparum* and *P.* vivax were observed. The most common malaria clinical manifestations were fever (96.5%), chills (91.3%), and headache (90.6%). Low back pain was present in almost half of the patients evaluated. The less frequent clinical manifestations were epistaxis (2.4%) and gingivorrhagia (1.7%). Paleness (59.2%) and choluria (38.3%) were also frequent in these patients regardless of the parasite species causing malaria. Twenty-nine (10.1%) patients presented hepatomegaly; it was more frequent in patients infected with *P. falciparum* than with *P. vivax*, although without significant differences between both species (10/69 [14.5%] and 16/200 [8%], respectively; *p* = 0.19). Splenomegaly (*p* = 0.02) and somnolence (*p* = 0.03) were associated with *P. falciparum* infection. Seizures and stupor only occurred in patients with *P. falciparum* infection (Table 2).

**Table 2:** Symptoms and signs of patients infected with malaria parasites

Symptoms/Signs	Total (N = 287; 100%)	*P. vivax* (N = 200; 69.7%)	*P. falciparum* (N = 69; 24%)	Mixed malaria (N = 18; 6.3%)	*p*-value
Symptoms					
Fever	277 (96.5)	193 (96.5)	66 (95.7)	18 (100)	0.49^*^
Chills	262 (91.3)	184 (92)	62 (89.9)	16 (88.9)	0.8^†^
Diaphoresis	127 (44.3)	95 (47.5)	27 (39.1)	5 (27.8)	0.16^†^
Otalgia	13 (4.5)	10 (5)	3 (4.3)	–	0.41^*^
Epistaxis	7 (2.4)	4 (2)	2 (2.9)	1 (5.6)	0.67^*^
Gingivorrhagia	5 (1.7)	3 (1.5)	2 (2.9)	–	0.56^*^
Dyspnea	32 (11.1)	24 (12)	6 (8.7)	2 (11.1)	0.75^†^
Cough	33 (11.5)	19 (9.5)	13 (18.8)	1 (5.6)	0.07^†^
Chest pain	34 (11.8)	26 (13)	7 (10.1)	1 (5.6)	0.56^*^
Abdominal pain	91 (31.7)	67 (33.5)	22 (31.9)	2 (11.1)	0.14^†^
Diarrhoea	39 (13.6)	29 (14.5)	7 (10.1)	3 (16.7)	0.6^†^
Emesis	54 (18.8)	33 (16.5)	16 (23.2)	5 (27.8)	0.28^†^
Myalgia	116 (40.4)	82 (41)	28 (40.6)	6 (33.3)	0.88^*^
Arthralgia	113 (39.4)	79 (39.5)	28 (40.6)	6 (33.3)	0.83^†^
Headache	260 (90.6)	182 (91)	61 (88.4)	17 (94.4)	0.69^†^
Low back pain	135 (47)	97 (48.5)	30 (43.5)	8 (44.4)	0.75^†^
Signs					
Jaundice	81 (28.2)	55 (27.5)	23 (33.3)	3 (16.7)	0.25^†^
Pallor	138 (48.1)	101 (50.5)	26 (37.7)	11 (61.1)	0.96^†^
Hepatomegaly	29 (10.1)	16 (8)	10 (14.5)	3 (16.7)	0.19^†^
Splenomegaly	14 (4.9)	5 (2.5)	7 (10.1)	2 (11.1)	0.02^*‡^
Oliguria	19 (6.6)	10 (5)	8 (11.6)	1 (5.6)	0.16^*^
Polyuria	42 (14.6)	29 (14.5)	11 (15.9)	2 (11.1)	0.87^†^
Choluria	110 (38.3)	77 (38.5)	27 (39.1)	6 (33.3)	0.9^†^
Somnolence	20 (7)	9 (4.5)	10 (14.5)	1 (5.6)	0.03^*§^
Stupor	3 (1)	–	3 (4.3)	–	–
Seizures	3 (1)	–	3 (4.3)	–	–
Oedema	7 (2.4)	3 (1.5)	3 (4.3)	1 (5.6)	0.32*
Ascites	1 (0.3)	–	1 (1.4)	–	0.23*

Data is expressed as numbers (percentage). *Fisher’s exact test; ^†^Chi-square; ^‡^*Post-hoc* analysis: significant difference for *P. falciparum* (standardized residue = 2.0); ^§^*Post-hoc* analysis: significant difference for *P. falciparum* (standardized residue = 2.4)

### Laboratory findings

Laboratory results are presented in Table 3. The average haemoglobin and haematocrit were lower in patients infected with *P. falciparum* than *P. vivax* (*p* < 0.05). Leukopenia was associated with *P. falciparum* infection (*p* < 0.05). Severe thrombocytopenia was more frequently observed in patients with *P. falciparum* (23.3% in *P. falciparum* versus 14.2% in *P. vivax*). However, the average platelet values ​​were within the range of moderate thrombocytopenia in all malaria patients, and no significant differences were observed between parasite species (*p* = 0.93). The mean glycaemia was lower in patients with *P. vivax* infection (*p* < 0.05), showing significant hypoglycaemia linked to *P. vivax* infection (*p* < 0.05). There were no differences between *P. vivax* and *P. falciparum* patients in their total bilirubin, aminotransferases (AST and ALT), and lactate dehydrogenase values (Table 3). No electrolyte-relevant abnormalities (sodium, potassium, and chlorine) or coagulation tests (PT and aPTT) were found.

**Table 3:** Paraclinical results according to infecting parasite

Paraclinical	Total (N = 287; 100%)	*P. vivax* (N = 200; 69.7%)	*P. falciparum* (N = 69; 24%)	Mixed malaria (N = 18; 6.3%)	*p*-value
Haemoglobin (g/dL)	10.99 (1.98)	11.24 (1.67)	10.33 (2.6)	11.12 (1.4)	0.03*^†^
Alterations (total)					0.041^‡^
Mild anaemia (12.9–11)	64 (39.8)	50 (47.2)	11 (25.6)	3 (25)	
Moderate anaemia (10.9–7)	68 (42.2)	40 (37.7)	21 (48.8)	7 (58.3)	
Severe anaemia (< 7)	6 (3.7)	2 (1.9)	4 (9.3)	–	
Haematocrit (%)	34 (6)	34.8 (5)	31.9 (8)	34.1 (3.9)	0.02*^†^
Leukocytes (x mm^3^)	6,290 (2,800)	6,230 (2,620)	6,390 (3,330)	6,410 (2,460)	0.93*
Alterations (total)	29 (18)	12 (11.3)	16 (37.2)	1 (8.3)	< 0.01^†||^
Leukopenia (< 4,000/mm^3^)	17 (10.55)	7 (6.6)	10 (23.26)	–	
Leukocytosis (> 10,000/mm^3^)	12 (7.45)	5 (4.72)	6 (13.95)	1 (8.33)	
Neutrophils (%)	47.3 (14.9)	46.3 (14.4)	48.3 (16.4)	50.6 (14.2)	0.52*
Lymphocytes (%)	47.1 (13.2)	47.6 (13.7)	46.1 (12.4)	46 (12.1)	0.78*
Platelets (x mm^3^)	89 (48)	88 (38)	90 (56)	93 (83)	0.93*
Alterations (total)	148 (91.9)	98 (92.5)	39 (90.7)	11 (91.7)	0.58^§^
Mild thrombocytopenia (< 150,000/mm^3^)	40 (24.8)	28 (26.4)	11 (25.6)	1 (8.3)	
Moderate thrombocytopenia (< 100,000/mm^3^)	81 (50.3)	55 (51.9)	18 (41.9)	8 (66.7)	
Severe thrombocytopenia (< 50,000/mm^3^)	27 (16.8)	15 (14.2)	10 (23.3)	2 (16.7)	
Glycaemia (mg/dL)	79.06 (24.9)	75.24 (22.4)	87.69 (28)	81.8 (27.7)	0.01*^†^
Hypoglycaemia (< 60 mg/dL)	39 (24.2)	32 (30.2)	4 (9.3)	3 (25)	0.02^§†^
Urea	34.83 (26.2)	33.49 (26)	37.74 (25.8)	36.29 (30.7)	0.64*
Creatinine (mg/dL)	1.1 (0.8)	0.9 (0.9)	0.96 (0.8)	0.81 (0.3)	0.72*
Elevation (> 3 mg/dL)	2 (1.2)	1 (0.8)	1 (3.6)	–	0.32^‡^

	*n* = 148 (100%)	*n* = 99 (66.8%)	*n* = 39 (26.4%)	*n* = 10 (6.8%)	
Total bilirubin [mg/dL]	2.17 [2]	2.45 [2]	2.3 [2]	1.8 [2]	0.39^¶^
Elevation (> 3 mg/dL)	57 (38.5)	40 (40.4)	15 (38.5)	2 (20)	0.45^§^
Direct bilirubin [mg/dL]	0.98 [1]	0.96 [1]	1.44 [2]	0.72 [1]	0.14^¶^
Indirect bilirubin [mg/dL]	1.32 [1]	1.42 [1]	1.31 [1]	0.8 [1]	0.39^¶^

	*n* = 146 (100%)	*n* = 99 (67.8%)	*n* = 37 (25.3%)	*n* = 10 (6.9%)	
AST (mg/dL)	100 (76)	99 (72)	100 (84)	109 (89)	0.92*
Elevation (> 35 IU/L)	124 (84.9)	85 (85.9)	31 (83.8)	8 (80)	0.86^§^
ALT [mg/dL]	77 [127]	94 [161]	51 [82]	37 [152]	0.12^¶^
Elevation (> 45 IU/L)	86 (58.9)	62 (62.6)	21 (56.8)	3 (30)	0.12^§^

	*n* = 144 (100%)	*n* = 100 (69.4%)	*n* = 35 (24.3%)	*n* = 9 (6.3%)	
LDH (IU/L)	538 (180)	529 (182)	552 (188)	581 (123)	0.61*
Elevation (> 250 IU/L)	131 (93.6)	91 (92.9)	31 (93.9)	9 (100)	0.52^‡^

Data for values are expressed as mean (SD–standard deviation–) except for Total, Direct, Indirect bilirubin, and ALT that are expressed as median [IQR –interquartile range–]. Alteration's data are expressed as numbers (percentages). *One-way ANOVA; ^†^*Post-hoc* analysis: significant difference between *P. falciparum* and *P. vivax* (*p* = 0.019; Tukey); ^‡^Fisher’s exact test; ^§^Chi-square; ^||^*Post-hoc* analysis: only significant for *P. falciparum* and leukopenia (standardized residue = 2.6); ^¶^Median’s test

### Severe malaria

According to the local and the WHO [3, 14] guidelines, patients were categorized as having severe or non-severe malaria. Among the 161 patients with complete clinical and paraclinical evaluation, 69 (42.9%) patients with a mean age of 33 years (SD 13) were classified as severe malaria cases. *Plasmodium vivax* infections caused 60.8% (42/69) of cases with at least one disease severity criteria. *Plasmodium falciparum* infection and mixed malaria infection caused 24/69 (34.7%) and 4.34% (3/69) severe cases, respectively. Proportionally, patients with *P. falciparum* infection were more likely to develop severe symptoms than *P. vivax* and mixed malaria infections (55.8% vs. 39.6% and 25%; *p* = 0.004). Although most severe malaria (88.4%) had one clinical complication, eight patients simultaneously presented two or more complications. The most frequent complication in patients with *P. vivax* was hyperbilirubinaemia (plasma or serum bilirubin > 3 mg/dL), and 38 of 42 patients (90.4%) presented it as the only criterion of severe malaria. In addition, there were four patients with two criteria: one with cerebral malaria and pulmonary oedema, one with kidney failure and severe anaemia, one with cerebral malaria and hyperbilirubinaemia, and one presenting severe anaemia and hyperbilirubinaemia. Similarly, the most frequent complication in patients with *P. falciparum* was hyperbilirubinaemia; 14 of 24 patients (58.3%) as the only criterion of severe malaria. However, six of the 24 patients (25%) presented cerebral malaria, and three (12.5%) had severe anaemia. Less frequent were spontaneous bleeding (2 of 24; 8.3%) and renal dysfunction (1 of 24; 4.1%). Among the patients with mixed infection, two presented hyperbilirubinaemia and one pulmonary oedema as criteria of severity. One patient with *P. vivax* and another with *P. falciparum* died due to cerebral malaria.

### Recurrences

A total of 285 (99.3%) patients were followed up by telephone; however, only 49.8% (142/285) completed the follow-up for three months. Thirty-five patients (24.7%) had a recurrence. Of these, 31 had only one new episode, and four had two new episodes during follow-up; most (65.7%) recurrences occurred one month after infection. The proportion of recurrences was similar in patients with *P. vivax* (25/199; 12.6%), *P. falciparum* (8/68; 11.8%), and mixed malaria (2/18; 11.1%) infections.

## Discussion

The dramatic increase in malaria transmission in Venezuela has changed the regional epidemiological landscape [1, 5]. Still, there is limited information about the current clinical profile of malaria cases in Venezuela. Although limited in its scope, this study offers a glimpse of the malaria patients' clinical characteristics among those seeking assistance in Ciudad Bolivar health centres, the Bolivar state's capital. Bolivar is a critical malaria hotspot in the region [1, 5]. As expected, 70% of the cases were caused by *P. vivax* as a single infection determined by microscopy. However, 76% of the enrolled patients have *P. vivax* if mixed malaria infections are included. Gold mining has been associated with malaria in the Bolivar state [5, 17, 19]; consistent with those findings, most patients in this study were young adults (17–35 years old; 51.6%), and the main occupation was gold mining (40.1%). However, this percentage differs from previous studies, where 115 of 162 (71%) malaria patients reported working in mines [20]. Those early reports focused on Sifontes, a municipality near the main mining area, whereas this study included a broader geographic region that likely changed the population targeted by the survey. Sifontes contributes 53.7% of malaria cases in the Bolivar state and 46.4% of the national incidence [20].

It has been hypothesized that human mobility to and from the mining areas has been a critical factor in the current malaria epidemic [5, 10, 11, 21–23]. Considering that most patients involved in gold mining do so out of the local legal framework (illegal miners), a close follow-up of the effect of such activities on malaria will require a prospective study that does not rely solely on self-reporting. Although this study cannot test the hypothesis that gold mining is an important driver of malaria transmission, the results presented here are consistent with the notion that gold mining is a malaria risk factor in this area [5] and likely, for other countries in the region [21–23]. Of interest is the significant association between the occurrence of *P. falciparum* malaria and Sifontes; this is consistent with the importance of this area with gold mining in this parasite incidence [5].

Malaria symptoms (fever, chills, and headache) [24, 25] were common, and signs such as skin-mucosa pallor, jaundice, and choluria were present in all patients regardless of the parasitic species. However, in contrast with other studies reporting low back pain in 3.7% [26] or 13.3% [27] of cases, this symptom was observed in almost half of the patients evaluated in this study. Notably, 10.1% (29/287) of the patients presented hepatomegaly. Hepatomegaly has been observed in endemic areas of Colombia (23–25%) [28–30], Indonesia (26.8%) [26], and previously in the Bolivar state (6.7%) [27]. The differences between Venezuela and these countries are a matter that requires additional investigation. It is also worth noticing that splenomegaly was more frequent in *P. falciparum* infections (10 of 69; 14.5%), whereas others reported this sign most frequently in *P. vivax* infected patients [24].

Although clinical manifestations commonly associated with malaria in the region were found [24, 31], some local differences cannot be explained with the data available. Whereas anaemia was not associated with parasite species in other studies emerging from the region, here, the mean haemoglobin was lower in patients with *P. falciparum* infection (*p* = 0.03) [24, 32]. Hypoglycaemia was associated with severe *P. falciparum* infection in previous reports [33–35], but it was observed in *P. vivax* infections in this study. A significant finding is that 69 of the 161 patients with complete clinical evaluations (42.9%) met at least one criterion for severe malaria as defined by the WHO. A similar result was reported in a small study in Merida state (western Venezuela) that registered 13 of 33 (39.3%) patients with some form of severe malaria [20]. This high prevalence contrast with findings from Colombia (0.47% to 7.5%) [24, 36] and Brazil (9%) [37] and are considered high compared to countries with intense malaria transmission, such as Papua New Guinea [38] and Indonesia [39], where severe malaria prevalence was 6.2% and 9%, respectively.

The high number of severe malaria cases could result from bias when studying patients seeking treatment in the city's main clinical centres. It can be speculated that, on average, the patients could have been suffering from more severe disease, forcing them to seek treatment in a major health centre. Nevertheless, as expected [40, 41], the relative frequency of severe cases was higher in patients with *P. falciparum* than *P. vivax* and mixed malaria cases. It is worth mentioning that because *P. vivax* infections are higher in prevalence, infections by this parasite are linked to the majority of severe cases in terms of absolute numbers. Thus, this study adds new evidence that *P. vivax* infections –considered benign malaria– can also result in severe disease [42–49].

Even considering the bias introduced by studying solely clinical cases, the observed high frequency of patients with at least one criterion of severe malaria is a matter of concern. Determining whether this pattern results from inadequate access to timely and effective treatment requires longitudinal studies with proper controls.

It is important to note that *P. vivax* patients with at least one severe malaria criterion also showed, on average, more complex infections. In particular, samples from 91 *P. vivax* infections were genotyped using nine microsatellite loci as part of another study [6]. An infection was considered multiclonal if it has more than one allele in at least one locus. The association between having a multiclonal infection with severe malaria events was tested. It was significant in *P. vivax* using a Fisher exact test (24 uncomplicated cases with single and 27 with multiple infections, ten severe malaria cases with single and 30 with multiple infections, *p* < 0.05). A similar analysis was not significant in 57 *P. falciparum*-infected patients with their samples genotyped with eight microsatellite loci. This pattern is consistent with a study conducted in Colombia [50], with *P. vivax* and *P. falciparum*. Although preliminary, given the limited number of samples with complete clinical evaluations, the finding suggests that this association should be further investigated in the region.

The patient's follow-up in this study relayed on self-reporting; still, the proportion of recurrences (24.7%) was similar to those reported elsewhere [51]. Although the treatment was provided in a clinical setting, it should not be considered supervised. Different factors have been linked to recurrences, such as a lack or poor adherence to treatment [52, 53], the use of inappropriate doses, the geographic location [54], and therapeutic failure of primaquine as a result of patients with CYP2D6 mutations [55, 56]. Measuring the differential contributions of these and other factors was outside the scope of this descriptive study. Furthermore, in patients experiencing recurrence is complicated to distinguish recrudescence (i.e., *P. vivax* malaria due to treatment failure) from reinfection (i.e., due to new infectious mosquito bites) or relapse (i.e., due to reactivation of dormant parasites in the liver) in the absence of genomics tools [57, 58]. Finally, because the evaluation of recurrences was through telephone calls, there could be a recall bias.

The information reported here depicts a scenario where severe malaria seems relatively high, including several with *P. vivax* infections. However, the study has limitations imposed by the circumstances where the data was collected. First, it only included clinical patients passively diagnosed with malaria when they were seeking treatment. Thus, subclinical and submicroscopic infections were not included, and the study was likely biased toward patients with severe symptoms seeking attention in the primary diagnostic centres. Second, the study covered loosely defined endemic areas, so there is no accurate estimate of prevalence per location. Given the limited number of individuals from some localities, the data was aggregated. Third, only 161 out of 287 patients had clinical laboratory tests due to limited resources. Fourth, thick smears were used to diagnose malaria, as is the standard in Venezuela [14]; however, they are less sensitive than molecular methods. This limitation is mitigated because all patients were symptomatic, so usually, those have infections detectable by microscopy. It is worth noting that the absence of molecular confirmation may have introduced biases, mainly in estimates of mixed malaria infections found in 6.3% of the patients when molecular methods have detected up to 45.5% [6, 56]. Even when some severe cases could have been mixed malaria infections, the high prevalence of patients with at least one criterion of severe disease, particularly in *P. vivax*, should be a matter of concern.

## Conclusions

Malaria infections were more prevalent in young male adults practicing illegal gold mining in Bolivar State. Thus, like other studies in the region, the data presented here indicate that transmission in mining areas requires immediate attention in terms of research and policies. A high number of severe malaria cases with *P. vivax* malaria was observed. This finding should prompt comprehensive studies to identify the factors leading to this pattern. Whether severe disease could be avoided or diminished with timely diagnosis and effective treatment is a matter that needs to be explored in the context of longitudinal studies, which should use active detection to characterize subclinical infections. Such prospective studies should include genotyping tools to allow exploring the factors driving recurrences.

## Data Availability

All data generated or analysed during this study are included in the article.

## Abbreviations

WHO: World Health Organization
AST: Aspartate aminotransferase
ALT: Alanine aminotransferase
LDH: Lactate dehydrogenase
PT: Prothrombin time
aPTT: Activated partial thromboplastin time

## References

[1] WHO (2020). World malaria report 2020.

[2] Espinoza JL (2019). Malaria resurgence in the Americas: an underestimated threat. Pathogens.

[3] Grillet ME, Villegas L, Oletta JF, Tami A, Conn JE (2018). Malaria in Venezuela requires response. Science.

[4] Moreno JE, Rubio-Palis Y, Martínez ÁR, Acevedo P (2014). Evolución espacial y temporal de la malaria en el municipio Sifontes del estado Bolívar Venezuela, 1980–2013. Bol Mal Salud Amb..

[5] Grillet ME, Moreno JE, Hernández JV, Vincenti-González MF, Noya O, Tami A (2021). Malaria in Southern Venezuela: the hottest hotspot in Latin America. PLoS Negl Trop Dis.

[6] Pacheco MA, Forero-Peña DA, Schneider KA, Chavero M, Gamardo A, Figuera L (2020). Malaria in Venezuela: changes in the complexity of infection reflects the increment in transmission intensity. Malar J.

[7] Gabaldon A (1983). Malaria eradication in Venezuela: doctrine, practice, and achievements after twenty years. Am J Trop Med Hyg.

[8] Chowell G, Munayco CV, Escalante AA, McKenzie FE (2009). The spatial and temporal patterns of falciparum and vivax malaria in Perú: 1994–2006. Malar J.

[9] Arevalo-Herrera M, Quiñones ML, Guerra C, Céspedes N, Giron S, Ahumada M (2012). Malaria in selected non-Amazonian countries of Latin America. Acta Trop.

[10] Recht J, Siqueira AM, Monteiro WM, Herrera SM, Herrera S, Lacerda MVG (2017). Malaria in Brazil, Colombia, Peru and Venezuela: current challenges in malaria control and elimination. Malar J.

[11] PAHO (2018). Actualización Epidemiológica: Aumento de malaria en las Américas.

[12] INE. Proyección de la población al 30 de junio, según grupos de edad y municipio, 2014–2021. Instituto Nacional de Estadísticas; 2011. http://www.ine.gov.ve/index.php?option=com_content&view=category&id=98&Itemid=51.

[13] WHO (2014). Severe Malaria. Trop Med Int Health.

[14] MPPS, PAHO (2017). Pautas de tratamiento en Casos de Malaria: República Bolivariana de Venezuela, Ministerio del Poder Popular para la Salud 2017.

[15] WHO (2016). WHO Global Malaria Programme. Malaria terminology WHO/HTM/GMP/2016.6.

[16] Cáceres JL (2013). Récord de incidencia malárica en Venezuela. Bol Mal Salud Amb.

[17] Cáceres JL (2011). La Malaria en el estado Bolívar, Venezuela: 10 años sin control. Bol Mal Salud Amb.

[18] Pacheco C, Moreno J, Herrera F (2019). Molecular detection and species determination of malaria parasites. Venezuela Emerg Infect Dis.

[19] Oletta JF. Análisis del Reporte Mundial de malaria. 2018, y la grave epidemia de malaria en Venezuela. Estimaciones para 2018. Informe Especial. Caracas: Sociedad Venezolana de Salud Pública, Red Defendamos la Epidemiología Nacional, 2018.

[20] MPPS. Boletin Epidemiológico, Semana Epidemiológica N°52—25 al 31 de Diciembre de 2016. Caracas: Ministerio del Poder Popular para la Salud de Venezuela; 2016, 33.

[21] Louzada J, de Almeida NCV, de Araujo JLP, Silva J, Carvalho TM, Escalante AA (2020). The impact of imported malaria by gold miners in Roraima: characterizing the spatial dynamics of autochthonous and imported malaria in an urban region of Boa Vista. Mem Inst Oswaldo Cruz.

[22] da Cruzfranco V, Peiter PC, Carvajal-Cortés JJ, dos Santos Pereira R, Mendonça Gomes MdS, Suárez-Mutis MC (2019). Complex malaria epidemiology in an international border area between Brazil and French Guiana: challenges for elimination. Trop Med Health..

[23] Castellanos A, Chaparro-Narváez P, Morales-Plaza CD, Alzate A, Padilla J, Arévalo M (2016). Malaria in gold-mining areas in Colombia. Mem Inst Oswaldo Cruz.

[24] Arévalo-Herrera M, Lopez-Perez M, Medina L, Moreno A, Gutierrez JB, Herrera S (2015). Clinical profile of *Plasmodium falciparum* and *Plasmodium vivax* infections in low and unstable malaria transmission settings of Colombia. Malar J.

[25] Knudson-Ospina A, Sánchez-Pedraza R, Pérez-Mazorra MA, Cortés-Cortés LJ, Guerra-Vega ÁP, Nicholls-Orejuela RS (2015). Perfil clínico y parasitológico de la malaria por *Plasmodium falciparum* y *Plasmodium vivax* no complicada en Córdoba. Colombia Rev Fac Med.

[26] Johan A, Natalia A, Djauhari W, Effendi RF (2020). Clinical and hemoglobin profile of malaria patients in Karitas Hospital, southwest Sumba, period of year 2017. Indonesian J Trop Infect Dis.

[27] Tovar C, Tovar R, Sandoval M, Yary S (2018). Comportamiento clínico y de laboratorio de malaria por *Plasmodium falciparum*. Complejo Hospitalario Universitario Ruíz y Páez. Ciudad Bolívar, Estado Bolívar, Venezuela. 2003–2012. Bol Venez Infectol..

[28] Ramírez JF, Porras B, Borrero E, Martínez SP (2016). Factors associated with the severity and complication of patients with malaria hospitalized between 2009 and 2013 in three municipalities of Colombia, case-control study. Malar J.

[29] O'Brien AT, Ramírez JF, Martínez SP (2014). A descriptive study of 16 severe Plasmodium vivax cases from three municipalities of Colombia between 2009 and 2013. Malar J.

[30] Tobón-Castaño A, Giraldo-Castro C, Blair S (2012). [Prognostic value of clinical and parasitological signs for severe malaria in patients from Colombia](in Spanish). Biomedica.

[31] Muddaiah M, Prakash PS (2006). A study of clinical profile of malaria in a tertiary referral centre in South Canara. J Vector Borne Dis.

[32] Kulkarni VK, Agrawal K (2017). A study of clinical profile of malaria with special reference to complications and outcome. Int J Adv Med.

[33] Thien HV, Kager PA, Sauerwein HP (2006). Hypoglycemia in falciparum malaria: is fasting an unrecognized and insufficiently emphasized risk factor?. Trends Parasitol.

[34] Osonuga OA, Osonuga AA, Osonuga IO, Osonuga A, Derkyi KL (2011). Prevalence of hypoglycemia among severe malaria children in a rural African population. Asian Pac J Trop Dis.

[35] White NJ, Warrell DA, Chanthavanich P, Looareesuwan S, Warrell MJ, Krishna S (1983). Severe hypoglycemia and hyperinsulinemia in falciparum malaria. N Engl J Med.

[36] Chaparro-Narváez PE, Lopez-Perez M, Rengifo LM, Padilla J, Herrera S, Arévalo-Herrera M (2016). Clinical and epidemiological aspects of complicated malaria in Colombia, 2007–2013. Malar J.

[37] Dos-Santos JC, Angerami RN, Castiñeiras CM, Lopes SC, Albrecht L, Garcia MT (2014). Imported Malaria in a non-endemic area: the experience of the university of Campinas hospital in the Brazilian Southeast. Malar J.

[38] Genton B, D'Acremont V, Rare L, Baea K, Reeder JC, Alpers MP (2008). Plasmodium vivax and mixed infections are associated with severe malaria in children: a prospective cohort study from Papua New Guinea. PLoS Med.

[39] Barcus MJ, Basri H, Picarima H, Manyakori C, Sekartuti, Elyazar I, et al. Demographic risk factors for severe and fatal vivax and falciparum malaria among hospital admissions in northeastern Indonesian Papua. Am J Trop Med Hyg. 2007;77:984–91. 17984364

[40] Tobón A, Giraldo C, Pineros J, Arboleda M, Blair S, Carmona-Fonseca J (2006). Epidemiologia de la malaria falciparum complicada: estudio de casos y controles en Tumaco y Turbo, Colombia, 2003. Rev Bras Epidemiol.

[41] Luxemburger C, Ricci F, Nosten F, Raimond D, Bathet S, White NJ (1997). The epidemiology of severe malaria in an area of low transmission in Thailand. Trans R Soc Trop Med Hyg.

[42] Quispe AM, Pozo E, Guerrero E, Durand S, Baldeviano GC, Edgel KA (2014). *Plasmodium vivax* hospitalizations in a monoendemic malaria region: severe vivax malaria?. Am J Trop Med Hyg.

[43] Medina-Morales DA, Montoya-Franco E, Sanchez-Aristizabal VD, Machado-Alba JE, Rodríguez-Morales AJ (2016). Severe and benign *Plasmodium vivax* malaria in Emberá (Amerindian) children and adolescents from an endemic municipality in Western Colombia. J Infect Public Health.

[44] Alexandre MA, Ferreira CO, Siqueira AM, Magalhães BL, Mourão MP, Lacerda MV (2010). Severe *Plasmodium vivax* malaria, Brazilian Amazon. Emerg Infect Dis.

[45] Raposo CCBS, Santos JB, Santos GMCd, Gonçalves EdGdR, Silva ARd (2013). *Plasmodium vivax* malaria: related factors to severity in the State of Maranhão, Brazil. Rev Soc Bras Med Trop.

[46] Kochar DK, Das A, Kochar SK, Saxena V, Sirohi P, Garg S (2009). Severe *Plasmodium vivax* malaria: a report on serial cases from Bikaner in northwestern India. Am J Trop Med Hyg.

[47] Naing C, Whittaker MA, Nyunt Wai V, Mak JW (2014). Is Plasmodium vivax malaria a severe malaria: a systematic review and meta-analysis. PLoS Negl Trop Dis.

[48] Andrade BB, Reis-Filho A, Souza-Neto SM, Clarêncio J, Camargo LM, Barral A (2010). Severe *Plasmodium vivax* malaria exhibits marked inflammatory imbalance. Malar J.

[49] Baird JK (2013). Evidence and implications of mortality associated with acute *Plasmodium vivax* Malaria. Clin Microbiol Rev.

[50] Pacheco MA, Lopez-Perez M, Vallejo AF, Herrera S, Arévalo-Herrera M, Escalante AA (2016). Multiplicity of infection and disease severity in *Plasmodium vivax*. PLoS Negl Trop Dis.

[51] Douglas NM, Nosten F, Ashley EA, Phaiphun L, van Vugt M, Singhasivanon P (2011). *Plasmodium vivax* recurrence following falciparum and mixed species malaria: risk factors and effect of antimalarial kinetics. Clin Infect Dis.

[52] Grietens KP, Soto V, Erhart A, Ribera JM, Toomer E, Tenorio A (2010). Adherence to 7-day primaquine treatment for the radical cure of *P. vivax* in the Peruvian Amazon. Am J Trop Med Hyg.

[53] Cheoymang A, Ruenweerayut R, Muhamad P, Rungsihirunrat K, Na-Bangchang K (2015). Patients' adherence and clinical effectiveness of a 14-day course of primaquine when given with a 3-day chloroquine in patients with *Plasmodium vivax* at the Thai-Myanmar border. Acta Trop.

[54] Battle KE, Karhunen MS, Bhatt S, Gething PW, Howes RE, Golding N (2014). Geographical variation in *Plasmodium vivax* relapse. Malar J.

[55] Martin Ramírez A, Lombardia González C, Soler Maniega T, Gutierrez Liarte Á, Domingo García D (2020). Several *Plasmodium vivax* relapses after correct primaquine treatment in a patient with impaired cytochrome P450 2D6 function. Malar J.

[56] Dijanic C, Nickerson J, Shakya S, Dijanic A, Fabbri M (2018). Relapsing malaria: a case report of primaquine resistance. Case Rep Infect Dis.

[57] Popovici J, Pierce-Friedrich L, Kim S, Bin S, Run V, Lek D (2019). Recrudescence, reinfection, or relapse? A more rigorous framework to assess chloroquine efficacy for *Plasmodium vivax* malaria. J Infect Dis.

[58] Escalante AA, Ferreira MU, Vinetz JM, Volkman SK, Cui L, Gamboa D (2015). Malaria molecular epidemiology: lessons from the International Centers of Excellence for Malaria Research Network. Am J Trop Med Hyg.

